# From Stress to Substance Use Disorders: The Expanding Role of Microglia–Astrocyte Crosstalk in Neuroimmune and Glutamate Alterations in the Nucleus Accumbens

**DOI:** 10.3390/ijms27010385

**Published:** 2025-12-30

**Authors:** Liliana Marina Cancela, Bethania Mongi-Bragato, María Paula Avalos, Flavia Andrea Bollati

**Affiliations:** Instituto de Farmacología Experimental de Córdoba (IFEC-CONICET), Departamento de Farmacología Otto Orsingher, Facultad de Ciencias Químicas, Universidad Nacional de Córdoba, Córdoba X5000HUA, Argentina; bethania.mongi@unc.edu.ar (B.M.-B.); mavalos4@jh.edu (M.P.A.); fbollati@unc.edu.ar (F.A.B.)

**Keywords:** microglia, astrocytes, glutamate, Nucleus Accumbens (NAc), neuroimmune signaling, cocaine, substance use disorders (SUDs), stress

## Abstract

This review examines convergent neurobiological mechanisms linking stress and drugs that drive stress-induced drug-related behaviors. It first outlines the main theoretical frameworks explaining substance use disorders (SUDs), emphasizing vulnerability factors—particularly stressful life events—that increase addiction risk. The analysis integrates preclinical evidence demonstrating that chronic stress facilitates cross-sensitization to psychostimulants and accelerates drug self-administration, underscoring how stress and drugs converge on glutamatergic and dopaminergic transmission within the Nucleus Accumbens (NAc). Special attention is given to the glial cells, particularly microglia and astrocytes, in mediating stress-induced neuroimmune activation and glutamate dysregulation in the NAc. Three major themes related to microglia–astrocyte crosstalk are addressed: (i) the contribution of these glial cells to neuroimmune and glutamatergic alterations induced by stress; (ii) their role in synaptic and structural plasticity changes within the NAc; and (iii) the mechanisms by which stress and drug exposure reshape glial–neuronal communication, driving the comorbidity between stress and SUDs. A dedicated section focuses on key neuroimmune signaling pathways—particularly the TNF-α/NF-κB axis—and their involvement in stress-induced vulnerability to cocaine addiction. Finally, the review discusses preclinical evidence supporting the therapeutic potential of repurposed glutamate-modulating agents as promising pharmacological candidates for treating comorbid stress and cocaine-use disorder.

## 1. Introduction: A Neurobiological Framework for the Comorbidity of Stress and Substance Use Disorders (SUDs)

Stress is a physiological and psychological state that arises when environmental demands are perceived to exceed an individual’s capacity to cope. While the stress response is inherently adaptive, enabling the organism to manage acute challenges effectively, its chronic or unpredictable activation imposes significant bioenergetics and neurobiological costs. Prolonged engagement of the stress response can shift the brain from a state of adaptive regulation to a maladaptive and pathophysiological condition, thereby increasing vulnerability to psychiatric disorders, including substance use disorders (SUDs) [1,2].

SUDs are severe neuropathological conditions representing the culmination of a series of neurobehavioral events that define the addictive process. This trajectory involves a progression from voluntary, controlled drug use to a gradual loss of control, culminating in compulsive drug-seeking behavior. A defining feature of addiction is the persistent vulnerability to relapse, even after extended periods of abstinence [3,4]. This enduring, relapsing nature reflects long-lasting neurobiological alterations within the reward circuit, often accompanied by cognitive impairments, motivational disturbances, and physiological adaptations that drive continued drug use despite adverse consequences [5]. From a diagnostic perspective, the term addiction is now encompassed within the broader construct of SUDs. In 2013, the DSM-5 unified the previously distinct diagnoses of substance abuse and substance dependence into a single continuum, defining SUDs as ranging from mild to severe based on the number of diagnostic criteria met.

Clinical evidence indicates that stressful life events are critical vulnerability factors for the development of SUDs, with a high degree of comorbidity observed between drug use and stressful or traumatic experiences [6]. Between 50% and 65% of individuals with post-traumatic stress disorder (PTSD) also suffer from SUDs, with a prevalence three to five times higher than in the general population [7,8]. Traumatic experiences are not only major risk factors for initiating SUDs but also potent triggers for relapse [9]. Preclinical studies using drug self-administration models have demonstrated that stress exposure facilitates drug-taking behavior [10,11]. Moreover, reinstatement models have demonstrated that stress, drug exposure, and conditioned cues can each independently trigger the reinstatement of heroin- or cocaine-seeking behavior in animals previously trained and subjected to extinction procedures [12,13]. These findings suggest that stress-, drug-, and cue-related stimuli converge on shared neural circuits that drive drug-seeking behavior.

Historically, addiction research has focused predominantly on neuronal adaptations [14,15,16], often neglecting the critical role of glial cells. However, it is increasingly recognized that glial-mediated dysregulation of glutamate homeostasis, neuroinflammation, and maladaptive synaptic remodeling within the mesocorticolimbic system plays a central role in the molecular and cellular processes underlying stress-induced drug-seeking, drug-taking, and relapse behaviors [17]. Integrating glial mechanisms into the neurobiological framework of stress-SUD comorbidity is thus essential for advancing our understanding of these overlapping psychiatric and addictive conditions and for developing novel therapeutic strategies that specifically target glial dysfunction.

An expanding body of clinical and experimental evidence further demonstrates that neuroadaptations triggered by chronic, uncontrollable stress, especially traumatic stress capable of inducing PTSD, and repeated drug exposure converge within the mesocorticolimbic system, a core component of the addiction circuitry. These shared pathological mechanisms reflect the profound impact of both stress and drugs on neural systems governing reward, emotional regulation, and executive control [18,19,20]. A hallmark of these alterations is the disruption of top-down control exerted by the prefrontal cortex over limbic and striatal regions. Dysfunction within this circuitry contributes to maladaptive behavioral phenotypes, including heightened impulsivity, emotional dysregulation, and compulsive drug-seeking, core features of both stress-related disorders and addiction [21,22]. Given that neuronal activity within the mesocorticolimbic system is tightly regulated by glial cells, understanding astrocyte and microglia function is essential to explain how stress and drug exposure remodel this circuitry.

Importantly, chronic stress is known to induce profound structural and functional changes in astrocytes and microglia [23]. While traditionally viewed as support cells, it is now evident that astrocytes and microglia actively regulate neuronal function by modulating glutamatergic neurotransmission, synaptic plasticity, and neuroimmune signaling [24]. Both chronic stress and exposure to addictive drugs produce overlapping alterations in glial activity, indicating that glial dysregulation may represent a central mechanism underlying the dual pathology of stress-related disorders and SUDs [17]. Of particular interest is the emerging evidence of astrocyte–microglia crosstalk mediated by cytokines such as interleukin 1-beta (IL-1β), Tumor necrosis factor-alpha (TNF-α), and complement protein C1q, which can reshape neuronal circuits under both physiological and pathological conditions, including those induced by chronic stress and/or drug exposure [25,26,27].

This review focuses primarily on psychostimulants, particularly cocaine, as they represent the most extensively studied class of drugs in preclinical models addressing stress-induced glutamatergic and neuroimmune alterations. Central to this discussion is the Nucleus Accumbens (NAc), a key hub within the motivation circuitry, where converging neurobiological and immune-related adaptations emerge in response to both stress and drug exposure. Throughout the review, we adopt a mechanistic perspective to examine stress–drug comorbidity, with particular emphasis on the proactive effects of stress in promoting drug-related behaviors. Specifically, we highlight its role in facilitating the acquisition of drug self-administration and in enhancing sensitivity to the psychostimulant effects of cocaine through cross-sensitization. This focus is guided by the depth and consistency of available preclinical evidence in these areas. Accordingly, although models investigating stress-induced reinstatement of drug-seeking behavior are not explored in detail, they are referenced when relevant findings contribute to the broader conceptual framework.

## 2. Stress as Vulnerability Factor in Substance Use Disorders (SUDs): Theoretical Approaches and Interacting Risk Factors

### 2.1. Vulnerability Factors

The development of SUDs results from a complex interaction between multiple vulnerability factors that predispose individuals to compulsive drug use. Evidence from preclinical, clinical, and epidemiological studies has identified a range of risk domains, including genetic predisposition, neurobiological traits, psychological characteristics, comorbid psychiatric disorders, and environmental, developmental, and social factors that shape an individual’s susceptibility to addiction [19,28,29,30,31]. Among these, environmental stressors, particularly exposure to stressful life events, have been consistently identified as major contributors to increased risk of drug use and the development of SUDs in humans [9,32,33,34,35,36,37,38]. Stress not only increases the probability of initial drug use but also exacerbates the transition to compulsive use and heightens the risk of relapse. Moreover, the persistent vulnerability to relapse even after extended periods of abstinence suggests that addiction is maintained not only by neuroadaptations resulting from repeated drug exposure, but also by enduring interactions between genetic factors, stress responsiveness, and environmental drug-associated cues [3,39]. These findings underscore the importance of considering stress not simply as a trigger but as a core component in the development and maintenance of SUDs.

### 2.2. Theoretical Approaches

A central issue in addiction neuroscience is understanding why, despite the widespread use of psychoactive substances, only 10–15% of individuals develop a compulsive, addiction-like pattern of drug use [40]. This observation has prompted the development of two major theoretical perspectives: the drug-centered and individual-centered models of addiction. The drug-centered perspective posits that addiction arises primarily as a consequence of the drug’s neurobiological impact. According to this view, repeated drug exposure induces long-lasting alterations in brain function, such as tolerance, dependence, behavioral sensitization, conditioned reinforcement, and withdrawal, which collectively shift the individual toward compulsive drug-seeking and drug-taking behavior [14,41,42]. These neuroadaptations are proposed to underlie not only the initiation and escalation of drug use, but also the persistence of addiction and the high probability of relapse after abstinence [15,16,43].

In contrast, the individual-centered perspective emphasizes the role of biological predispositions—shaped by genetic, developmental, and environmental factors, such as stress exposure—that interact to determine an individual’s susceptibility to addiction [44,45]. This model is supported by extensive preclinical data showing individual differences in vulnerability to psychostimulant self-administration, including strain-specific differences in drug-seeking behavior. These behavioral differences have been correlated with drug intake during the acquisition phase, suggesting a trait-like predisposition to compulsive use.

### 2.3. Emergent Integrative Perspective of Substance Use Disorders (SUDs)

Beyond the direct reinforcing properties of drugs, it is well-established that chronic drug use leads to long-lasting dysregulation of the brain’s reward system, resulting in the emergence of an anti-reward state [46]. This state is characterized by heightened stress responsiveness and a diminished capacity to experience reward, which together promote negative emotional states that drive continued drug use and relapse. In this framework, stress plays a central role at all stages of the addiction cycle, from initiation to maintenance and relapse. However, despite its importance, the molecular and neurophysiological mechanisms that mediate the interaction between stress and addiction, particularly in the context of comorbid conditions such as PTSD and SUDs, are not yet fully understood. A growing body of research is now dedicated to clarifying how stress-related molecular pathways contribute to the maladaptive reorganization of reward circuits, and how genetic and epigenetic factors modulate this vulnerability [47,48].

Emerging evidence indicates that life experiences, including exposure to chronic stress, can produce long-lasting changes in the brain’s epigenetic architecture, thereby altering gene expression patterns that influence vulnerability to addiction. These epigenetic modifications may sensitize neural circuits to drug exposure, facilitating the transition from voluntary use to compulsive drug-seeking behavior [49]. Complementing this framework, recent transcriptomic studies have characterized the molecular adaptations that occur after exposure to drugs of abuse. Browne et al. (2023) [50] mapped heroin-induced transcriptional changes across the brain’s reward circuitry, uncovering gene networks linked to drug intake, seeking, and relapse. Similarly, Mews et al. (2023) [47] characterized cocaine-related transcriptional alterations, revealing both overlapping and distinct molecular profiles compared to opioid-use disorder. Importantly, the gene expression signatures identified in both studies show strong correspondence with human data, reinforcing the translational relevance of preclinical models and suggesting common molecular targets for future interventions. Together, these findings support a model in which experience-driven epigenetic modifications create a permissive state for addiction vulnerability, while drug exposure produces additional transcriptional reorganization that reinforces drug-seeking behavior.

Taken together, these findings support the view that SUDs are complex, multifactorial conditions that cannot be understood solely as consequences of chronic drug exposure. Instead, they emerge from a dynamic interplay between genetic predispositions, phenotypic traits, environmental stressors—particularly chronic or repeated stress experienced in daily life—and drug-induced neuroplasticity [3]. From this perspective, stress emerges as a pivotal factor that interacts with individual genetic background to shape early drug responses. These initial responses may trigger neuroadaptive processes that, when sustained by ongoing psychological and environmental pressures, significantly increase the risk of developing compulsive drug use. Although a detailed analysis of genetic and epigenetic contributions lies beyond the scope of this review, it is important to acknowledge that gene–environment interactions likely contribute to individual vulnerability and warrant further investigation. Here, our primary objective was to examine the enduring neuroadaptations through which stress modulates drug action within the mesolimbic reward circuitry, while situating genetic factors within a broader conceptual framework. This integrative perspective may help explain why only a subset of individuals transitions from drug use to addiction and underscores the need for personalized prevention and treatment strategies that consider both neurobiological and environmental determinants of addiction risk.

## 3. Motivational Neural Circuits Implicated in Stress and Substance Use Disorders (SUDs)Vulnerability

**Figure 1:**
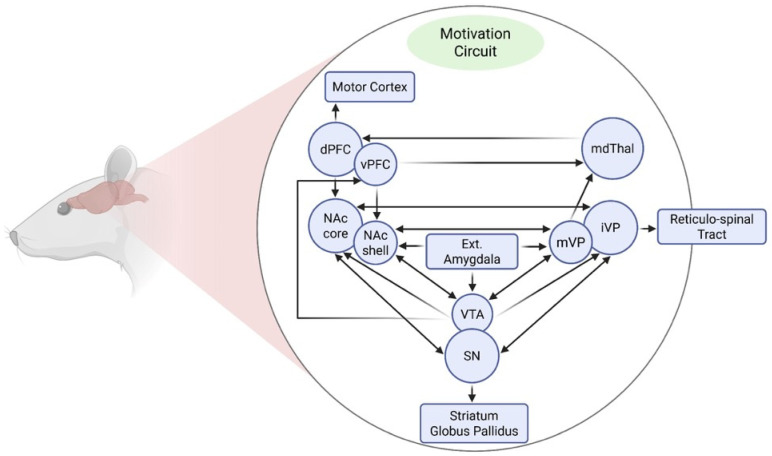
The motivation circuit. This panel presents the organization of the motivation circuit, detailing the key nuclei and the directional projections that link limbic processing with pyramidal and extrapyramidal motor outputs. As illustrated, the NAc core functions as a central hub where reward- and aversion-related information converges. Motivational states are translated into action through the integration of inputs from regions such as the amygdala, hippocampus, and prefrontal cortex, with DA contributing to the assignment of motivational significance to goals. VP: ventral pallidum; PFC: prefrontal cortex; SN: substantia nigra; VTA: ventral tegmental area; Thal: thalamus. Modified from [55]. This figure was created by BioRender.com.

From a neurobiological perspective, mesencephalic dopaminergic neurons projecting from the Ventral tegmental area (VTA) to the NAc and various cortical regions constitute a key substrate modulated by stress-related hormones, such as corticotropin-releasing factor (CRF) and glucocorticoids. During stressful experiences, these hormones are released and potently enhance dopaminergic transmission [45,51]. Activation of this mesocorticolimbic pathway (Figure 1) mediates the reinforcing properties of drugs of abuse [52]. Consequently, stress-induced alterations in this system can facilitate the initiation and maintenance of drug self-administration [45,53,54]. At the molecular level, exposure to various stressors or repeated drug administration activates both glutamatergic and dopaminergic systems, triggering intracellular signaling cascades that alter gene expression within motivational circuits [55,56]. These transcriptional changes promote long-lasting neurochemical and structural adaptations underlying neural sensitization, a process that manifests behaviorally as psychomotor sensitization [14,57]. Notably, psychomotor sensitization is tightly linked to enhanced drug self-administration and relapse vulnerability [57,58].

As noted by Pierce and Kalivas (1997) [55], and by Scofield et al. (2016) [59] more recently, the NAc receives convergent glutamatergic inputs from the cortical, allocortical, thalamic, midbrain, and brainstem regions, and projects to basal ganglia structures including the ventral pallidum and substantia nigra pars reticulata, as well as to mesencephalic, hypothalamic, and extended amygdala areas. Afferent projections often innervate both the core and shell of the NAc along defined topographic gradients (e.g., dorsoventral hippocampal projections terminate from lateral to medial regions of the NAc). Within this circuit, dopaminergic, glutamatergic, GABAergic, endocannabinoid, and opioid signaling systems interact in complex and dynamic ways. In particular, the mesocorticolimbic and nigrostriatal dopamine (DA) pathways underlie both voluntary motor function and motivated behaviors, and they are co-opted during drug exposure to drive compulsive drug seeking and consumption. Furthermore, Nall et al., (2021) [60] pointed out that distinct circuit motifs have been implicated in specific aspects of drug-seeking behavior, including: (i) an Action/Reward subcircuit comprising the NAc, ventral pallidum, and VTA; (ii) a Prefrontal subcircuit involving the prelimbic, infralimbic, and insular cortices; (iii) a stress subcircuit encompassing the central amygdala and bed nucleus of the stria terminalis; and (iv) a Diencephalic subcircuit involving the lateral hypothalamus. While the precise contributions of the NAc shell, insular cortex, and ventral pallidum remain partially unresolved, extensive evidence across species supports a critical role for these structures in mediating both drug- and natural-reward seeking.

The NAc itself—a key target of both stress and drugs of abuse—is subdivided into two functionally distinct compartments: the core and the shell [61,62], with differential anatomical afferents and efferent projections [63], which may account for different aspects of the drug rewarding process [64]. The shell plays a critical role in assigning salience to motivationally relevant stimuli and is particularly involved in the formation of stimulus–reward associations, especially during early phases of drug conditioning [65,66]. In contrast, the core is essential for sensorimotor integration [67] and supports the expression of learned behaviors elicited by cues predicting salient events [62,68]. It is also a central node for the long-term expression of drug- and stress-induced sensitization and for cue-triggered drug-seeking behavior [64,69,70]. In this context, glutamatergic projections from the prelimbic prefrontal cortex to the NAc core are critical for the enduring control of cocaine seeking and reinstatement [71].

Physiologically, the NAc integrates pharmacological and environmental stimuli to generate adaptive or maladaptive behavioral responses [55,72,73]. Natural rewards such as food, sex, and social interaction increase DA levels in the NAc, eliciting hedonic states that reinforce survival-related behaviors. Drugs of abuse, however, usurp this system, inducing supraphysiological DA release that is both more intense and more sustained than that produced by natural reinforcers [74,75,76]. Although this drug-evoked DA surge lacks adaptive value for survival, it nevertheless powerfully recruits reinforcement mechanisms and remodels neural circuits through maladaptive forms of plasticity. Despite differing pharmacological targets, chronic exposure to addictive substances—including alcohol, nicotine, opioids, cannabinoids, and psychostimulants—converges on the dysregulation of the mesocorticolimbic system, resulting in persistently elevated extracellular DA levels in the NAc [77,78]. This dopaminergic hyperactivity contributes to both the heightened reinforcing properties of drugs and the exaggerated locomotor responses characteristic of psychostimulant exposure [79].

These interconnected systems form the neurobiological foundation of addiction. Their modulation by stress underscores the critical importance of understanding how environmental factors interact with neural circuits to promote vulnerability to SUDs.

## 4. Neurobiological Mechanisms Linking Stress and Psychostimulants: Dopaminergic and Glutamatergic Interactions in the Nucleus Accumbens (NAc)

**Figure 2:**
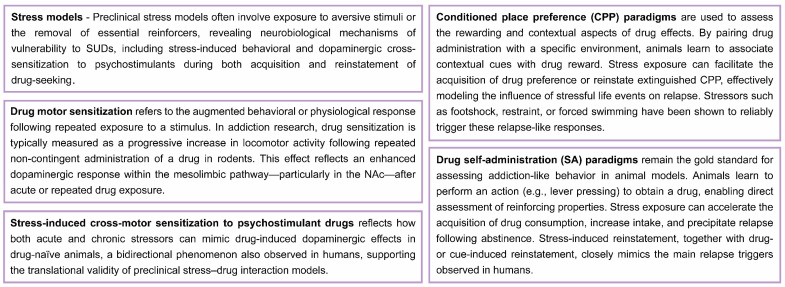
Preclinical paradigms of stress-induced vulnerability to SUDs.

Although the precise mechanisms by which stress promotes SUDs remain unclear [18,80], convergent preclinical evidence demonstrates that chronic stress reliably increases vulnerability to psychostimulant self-administration in rodents (see Figure 2) [81]. Specifically, exposure to stressors such as intermittent social defeat or restraint (acute or chronic) leads to long-lasting neuroadaptations—including locomotor and dopaminergic cross-sensitization—which result in escalated cocaine intake during extended-access sessions [82,83,84,85]. These findings are consistent with both clinical and preclinical observations indicating that certain forms of stress can promote the initiation, escalation, and/or relapse of drug use [30,86,87,88].

CRF and glucocorticoids (corticosterone in rodents) orchestrate many of the hormonal interactions between stress and drugs of abuse. Acting through CRFR1 and CRFR2, CRF is a major driver of stress-evoked drug-seeking and relapse [11,13,86,89,90,91,92]. Although CRF’s canonical role is to activate the hypothalamic–pituitary–adrenal axis [93], it also modulates extrahypothalamic nuclei—including the amygdala, bed nucleus of the stria terminalis and VTA—that govern motivation and reward [94,95]. The VTA serves as a major site of convergence where CRF and psychostimulants synergistically enhance DA release within mesolimbic and cortical circuits [96,97]. Dopaminergic neurons in the VTA, projecting to the NAc, prefrontal cortex, and amygdala, are pivotal for mediating both the acute reinforcing effects of drugs of abuse and the long-term neuroadaptations underlying addiction [98,99]. Within this circuitry, CRF signaling in the VTA promotes neuroadaptive alterations in dopaminergic activity through CRFR1 activation [100,101]. However, studies directly assessing the role of VTA CRF in regulating mesocortical DA output have yielded mixed results [102]. Strengthening this causal link, Refojo et al. (2011) [103] demonstrated that selective deletion of CRFR1 from VTA DA neurons markedly attenuated stress-induced elevations in prefrontal DA, underscoring the contribution of CRF-dependent mechanisms to stress-driven dopaminergic adaptations that may enhance addiction vulnerability.

In parallel, foundational rodent studies demonstrated behavioral cross-sensitization between stress and amphetamine exposure [104]. Subsequent research further revealed that individual differences in stress reactivity—such as novelty-induced locomotion—and prior drug experience synergistically increase vulnerability to the reinforcing effects of psychostimulants, including amphetamines and cocaine [105,106]. Importantly, corticosterone—the primary glucocorticoid in rodents—also emerges as a key hormonal mediator of this interaction. While sustained corticosterone signaling following stress enhances psychostimulant intake, adrenalectomy or physiological corticosterone replacement significantly attenuates the stress-induced potentiation of drug use [107,108,109]. Furthermore, a variety of stressors—including electric foot shock, restraint, social defeat, and food deprivation—promote cocaine self-administration, often through elevations in corticosterone [110,111].

The rewarding properties of these drugs, assessed via conditioned place preference [112,113,114], and their reinforcing efficacy in self-administration paradigms [45,115], are likewise amplified in stress–drug cross-sensitization paradigms. Social defeat stress similarly sensitizes animals to cocaine and amphetamine, promoting binge-like intake patterns [116,117], while environmental factors such as social housing conditions modulate the magnitude of stress-evoked drug seeking in mice [118]. Notably, when stress is experienced within the drug-taking context—for example, during intra-session electric footshock—animals show an escalation of cocaine intake that mirrors the excessive consumption observed in extended-access models of addiction [119,120].

Likewise to that observed following stress, repeated, non-contingent administration of cocaine, amphetamine, nicotine, ethanol, morphine, or Δ^9^-tetrahydrocannabinol (THC) reliably induces locomotor sensitization in rodents [121,122,123,124,125,126]. Although a single drug injection can elicit a transient increase in locomotor activity [127,128], stable, long-term sensitization generally requires repeated exposure. Likewise, acute or chronic stress exposure can cross-sensitize organisms to the psychomotor-activating and reinforcing effects of drugs of abuse. Social defeat, food restriction, foot-shock, prenatal stress, and restraint stress each enhance locomotor cross-sensitization to cocaine [11,13,84,129,130] and to amphetamine [131,132,133]. Crucially, repeated exposure to these stressors does not diminish but rather consolidates the sensitized response, underscoring the pathological significance of chronic, everyday stress. These findings point to overlapping neural substrates engaged by both stress and psychostimulant exposure. Consistent with this view, various stressors—foot-shock [134], restraint [51,131], and prenatal stress [135]—activate mesolimbic DA transmission and heighten locomotor responses to cocaine and amphetamine. Nevertheless, although stress and psychostimulants produce parallel changes in behavior and dopaminergic signaling, a direct causal link between these two domains has yet to be fully established [55,126,136,137].

Glutamatergic afferents tightly control VTA DA neurons and mediate both rapid and long-term adaptations [138]. In cocaine-experienced animals, CRF magnifies excitatory transmission onto these neurons [139]. In vitro, CRFR2 activation strengthens NMDA currents, underscoring glutamate as a common substrate for stress and psychostimulant action [140]. CRF-dependent amplification of AMPA drive further escalates mesocorticolimbic DA output [141]. Consistent with this, acute stress and diverse addictive substances—including cocaine, amphetamine, morphine, ethanol, and nicotine—increase the AMPA/NMDA ratio in VTA DA neurons [100]. Interestingly, direct CRF infusion into the VTA produces enduring synaptic plasticity via CRFR1, potentiating NMDA-receptor currents, upregulating AMPA-receptor function, and elevating the AMPA/NMDA ratio after stress or drug exposure [100,101,140,141]. At the same time, CRF diminishes GABA B and D2 receptor signaling, further boosting DA-neuron excitability [142].

Interestingly, stress not only increases extracellular glutamate concentrations in the VTA by enhancing synaptic glutamate release, but also exerts similar effects in other brain regions, including the medial prefrontal cortex, striatum, and NAc [143]. In parallel, both acute and chronic administrations of psychostimulants, such as cocaine and amphetamine, have been shown to elevate glutamate release in the VTA and NAc [144,145,146]. These effects are largely mediated by glutamatergic projections from the prefrontal cortex and amygdala to mesolimbic structures [147,148,149]. One of the key mechanistic implications of this corticolimbic glutamatergic activation is its ability to modulate the mesolimbic DA system, a process fundamental to the neurobiology of addiction [143,150]. Although the NAc is often treated as a single entity, accumulating evidence highlights functional divergence between its core and shell subregions in shaping behavioral and neurochemical responses to stress and drugs [64,69,70]. Pacchioni et al. (2007) [132] showed that a single restraint session potentiates amphetamine-evoked DA release in both compartments at 24 h, yet this effect endures for at least eight days only in the core, waning over time in the shell. Extending this dissociation, García-Keller et al. (2013) [130] found that acute restraint stress produces cross-sensitization to cocaine exclusively within the NAc core, where elevated dopaminergic responses persist up to 21 days post-stress. Chronic restraint stress produces a similar pattern: Avalos et al. (2022) [151] reported a sustained, stress-induced enhancement of cocaine-evoked DA release that is restricted to the NAc core, confirming a persistent core–shell asymmetry. This asymmetry is also reflected in structural remodeling, with an increase in mushroom spine density observed in the NAc core but not in the shell, following chronic restraint stress.

Glutamatergic mechanisms are central to this behavioral phenomenon of cross-sensitization between stress and psychostimulants (cocaine and amphetamine). For instance, pharmacological blockade of NMDA or AMPA receptors reverses stress-induced behavioral sensitization to cocaine and amphetamine, respectively [130,132]. Acute restraint elevates AMPA-receptor surface expression in the NAc core, heightening sensitivity to intra-NAc AMPA injection and to cocaine-induced locomotion—effects abolished by NMDA or AMPA receptor antagonists [130]. Likewise, chronic stress induced cross sensitization to cocaine psychostimulant effect and escalating drug-self administration along 10 days. Furthermore, it correlates with increased AMPA-receptor expression and structural plasticity—namely, greater mushroom-type spine density—within the core [84,129,151,152]. Together, these findings underscore a persistent, core-specific glutamatergic remodeling that underlies stress-induced vulnerability to psychostimulant effects (see details of synaptic plasticity in Section 8).

## 5. Dysregulation of Glutamate Homeostasis in the Nucleus Accumbens (NAc) Core as a Key Mechanism of Stress-Induced Cocaine Vulnerability

Repeated stress exposure is thought to produce a persistent hyperglutamatergic state within key nodes of the reward circuit, such as the NAc, thereby facilitating the development of behavioral sensitization and drug-seeking behavior [84,92,129,130,151,153,154]. A central mechanism underlying these adaptations involves the downregulation of the glutamate transporter 1 (GLT-1) in the NAc core, a pathogenic hallmark proposed to mediate the heightened vulnerability to addictive behaviors induced by drugs, and by acute or chronic restraint stress exposure [21,84,130].

It is well-established that cocaine-evoked increases in glutamate release within the NAc core occur exclusively in sensitized animals, but not in non-sensitized controls or saline-treated subjects after prolonged withdrawal [145,155]. Similar results have been reported in the VTA, where Kalivas and Duffy, (1998) [156] showed that a cocaine challenge selectively elevated glutamate release in previously sensitized animals after 21 days of withdrawal. Basal extrasynaptic glutamate concentrations in the NAc core critically regulates synaptic glutamatergic activity through presynaptic metabotropic glutamate receptors (mGluR2/3), which exert inhibitory feedback on glutamate release [157,158]. Notably, following chronic cocaine exposure, withdrawal reduces basal extracellular glutamate levels in the NAc core [159], while cocaine re-exposure elicits a marked glutamate surge [145,155]. These adaptations have been linked to impaired cystine–glutamate exchange via system Xc^−^ and decreased GLT-1-mediated uptake [159,160,161]. Consistently with this, current evidence indicates that most extracellular glutamate detected by microdialysis originates from astrocytic mechanisms rather than vesicular neuronal release [158,162,163,164]. Compelling evidence links both stress and psychostimulant exposure to persistent impairments in glutamate clearance, particularly via the downregulation of astrocytic GLT-1 across motivation-related brain regions. For example, GLT-1 decreases have been observed in the hippocampus and cortex [165] and in corticolimbic slices following inescapable footshock [166], as well as in the prefrontal cortex and striatum after chronic social defeat stress [167]. Consistent findings have emerged from addiction models, in which prolonged self-administration and withdrawal from cocaine, amphetamine, heroin, nicotine, and ethanol result in robust GLT-1 reductions, particularly in the NAc core [160,161,168,169,170,171,172,173,174]. Fischer-Smith et al. (2012) [175] further demonstrated that this downregulation persists throughout cocaine abstinence and is most pronounced in the NAc core, regardless of prior drug intake levels.

Consistently, both acute and chronic restraint stress lead to long-lasting disruptions in glutamate homeostasis within the NAc core. Using the no-net-flux microdialysis method, elevated basal glutamate concentration was detected in the NAc core—but not the shell—up to 21 days after the first restraint session (acute restraint stress: García-Keller et al., 2013 [130]; chronic restraint stress: Avalos et al., 2022 [151]). Importantly, this imbalance contributed to long-term postsynaptic neuroadaptations within the NAc core and to associated behavioral consequences. Specifically, chronic restraint stress promoted cross-sensitization to cocaine and facilitated the acquisition of cocaine self-administration when assessed 21 days after the initial restraint stress exposure (2 h daily for seven consecutive days) [85,129,152,176]. Remarkably, even a single 2 h restraint session was sufficient to induce behavioral sensitization and enhance cocaine self-administration [84]. Critically, treatment with the β-lactam antibiotic ceftriaxone, which upregulates GLT-1 expression, reversed both the stress-induced behavioral alterations and the associated GLT-1 downregulation. It should be noted that acute 2 h restraint stress did not affect Na^+^-independent ^3^H-glutamate uptake, which estimates cystine-glutamate exchanger activity, but significantly reduced Na^+^-dependent uptake, which reflects GLT-1 function. This reduction in glutamate uptake was further supported by whole-cell patch-clamp recordings of NAc core medium spiny neurons (MSNs), demonstrating greater synaptic glutamate spillover in stress compared with control animals [84]. Together, these findings support the notion that stress-induced dysregulation of glutamate homeostasis via GLT-1 in the NAc core is a central mechanism driving increased sensitivity to the psychomotor and reinforcing effects of cocaine. In line with this, Guzman et al. (2021) [114] showed increased NAc core glutamate levels associated with a cocaine-paired context during restraint stress-induced reinstatement of extinguished cocaine-conditioned place preference in rats.

GLT-1 is strategically localized on astrocytic processes adjacent to the synaptic cleft, enabling rapid and efficient clearance of synaptically released glutamate to maintain extracellular homeostasis [177,178]. Disruption of GLT-1-mediated uptake following restraint stress results in glutamate spillover into the extrasynaptic space, leading to two key pathophysiological consequences: (i) activation of mGluR2/3 and loss of GLT-1’s neuroprotective constraint against excessive postsynaptic glutamate stimulation, and (ii) a prolonged decay time of NMDA receptor-mediated currents in the NAc core, detectable up to 21 days after stress, which strengthens and prolongs glutamatergic transmission in this nucleus [84]. Given the predominantly perisynaptic localization of NR2B subunits [173], these findings provide strong support for the glutamate spillover hypothesis. Notably, ceftriaxone treatment reverses this stress-induced increase in NMDA decay time within the NAc core. This electrophysiological evidence derives from a collaborative study between Peter Kalivas’s laboratory and our own [84]. Consistent with earlier reports [160,161,179], more recent evidence demonstrate that ceftriaxone also normalizes basal extracellular glutamate levels in the NAc core [84,85,151], thereby reestablishing mGluR2/3-mediated inhibitory feedback and preventing the stress-induced behavioral consequences.

Moreover, the persistent elevation in basal extracellular glutamate resulting from reduced GLT-1 function contributes to maladaptive postsynaptic changes, including structural plasticity in MSNs [84,85,129,151] (see Section 8 for further details). Together, these findings highlight astrocytic GLT-1 dysregulation as a unifying mechanism underlying stress and drug-induced glutamatergic maladaptations within the reward circuit, ultimately predisposing individuals to SUDs.

## 6. Glial Cells in the Nucleus Accumbens (NAc): Microglia and Astrocytes Characteristics and Physiological Roles in Glutamate and Neuroimmune Regulation

Glial cells, once considered merely immune sentinels or passive structural elements, are now recognized as active regulators of neuronal function, particularly in the NAc, where they modulate glutamatergic transmission, neuroimmune signaling, and synaptic plasticity [180]; this section provides an overview of their defining characteristics and physiological functions in this brain area.

### 6.1. Microglia: Characteristics, and Funtionnal Adaptations

Microglia account for approximately 10–20% of the total brain cell population, with a higher density in gray matter than in white matter [181]. As the brain’s resident macrophages, constituting the innate immune defense of the central nervous system (CNS) [182,183], microglia express a wide array of receptors, including those for neurotransmitters (glutamate, GABA, acetylcholine, DA, norepinephrine), neuromodulators (cannabinoids, opioids), pattern recognition molecules (e.g., TLR2, TLR4), purinergic receptors, glucocorticoid and mineralocorticoid receptors, and receptors for neurotrophins, cytokines, chemokines, complement proteins, and CSF-1 [184], enabling them to detect and integrate a wide range of signals relevant for NAc function.

Even in their highly ramified “resting” state, microglia exhibit continuous motility, extending and retracting processes to monitor the local environment without moving their soma or disrupting neuronal circuitry [185,186,187]. This constant surveillance reflects a vigilant immunological state, which extends beyond immune functions to support microglial involvement in circuit refinement and plasticity within motivational networks. Thus, microglia have a physiological role within the NAc, where they participate in synaptogenesis and activity-dependent synaptic pruning under normal conditions. They eliminate synapses during development [188,189] and modulate cognitive functions such as learning and memory [190,191]. Notably, synaptic pruning in the NAc during adolescence exhibits sex-specific patterns, targeting distinct synaptic inputs in males and females [192]. Microglial elimination of DA receptors during adolescence influences sex-specific development of NAc circuitry and social behavior. Supporting this, Csf1rΔFIRE/ΔFIRE mice lacking microglia show reduced excitatory synapse formation, altered presynaptic release, and changes in postsynaptic kinetics, underscoring their essential role in sculpting neural networks underlying adult behavior [193].

Microglia also interact directly with NAc synapses [194,195,196,197], facilitating bidirectional communication with neurons and maintaining synaptic homeostasis [198,199], influencing synaptic plasticity through the release of a variety of signaling molecules, including ATP [200], neurotransmitters [201,202,203,204], extracellular matrix components [205,206] and cytokines [207], some of which are involved in the astrocytic dependent mechanism of glutamate homeostasis [208,209,210,211,212,213]. In this sense, microglia also participate in glutamate signaling. Under specific conditions, they express the system Xc^−^ [214,215,216] and, although capable of expressing glutamate transporters like GLT-1, they account for only ~10% of glutamate uptake under physiological conditions [184]. Their transporter expression increases in pathological states, suggesting a compensatory role during glutamatergic dysfunction, and positioning them as complementary modulators of NAc excitatory balance [217,218,219].

Microglial populations in the NAc can be identified by specific markers, including CSF-1R (CD115), CX3CR1, CD68, F4/80, CD11b, and Iba-1. The expression of CD11b and Iba-1 is often upregulated upon activation [220,221,222], making them useful tools for assessing microglial states in physiological and pathological conditions. Such phenotypic distinctions are relevant because microglia, once considered mere sensors of injury [182], are now known to actively initiate and propagate brain pathologies. Their activation can be triggered by diverse stimuli, including glutamate [24], and is a graded context-dependent process, rather than a binary switch, varying across multiple brain regions according to microenvironmental cues [223,224]. Microglial morphological changes associated with activation include process retraction, soma hypertrophy, and transformation into an amoeboid shape [225]. Intermediate forms, like hyper-ramified microglia, also occur [186,187,226]. Although the functional implications of this hyper-ramified morphology are not fully understood, it is generally considered an intermediate state between resting and fully activated microglia, often interpreted as an early stage of hypertrophy. This phenotype may reflect subtle homeostatic shifts [226] or responses to non-pathological, experience-dependent stimuli [194,227,228,229,230].

Importantly, activated microglia can return to a resting-like morphology while retaining epigenetic changes that leave them in a “primed” state [231,232]. These “experienced” microglia exhibit exaggerated responses to subsequent stimuli and may contribute to the etiology of neuropsychiatric conditions involving glial dysfunction.

As mentioned, microglial functions are closely integrated with astrocytes. The following section therefore focuses on astrocytes, detailing their neurophysiology and roles in synaptic regulation within stress- and drug-sensitive brain circuits.

### 6.2. Astrocytes: Characteristics, Neurotransmitter Regulation, and Immune Functions

Astrocytes are the predominant non-neuronal cells in the CNS, outnumbering neurons by approximately ten to one and accounting for more than 50% of all CNS cells [233]. In subcortical regions, including the NAc, they represent about 20% of the total cellular population [234,235,236].

Far from being passive support cells, astrocytes participate in a broad spectrum of critical functions, including formation and regulation of the blood–brain barrier, protection against excitotoxicity, promotion of synaptic plasticity, coordination of neural network activity, metabolic support, and participation in bidirectional communication with neurons, other astrocytes, and microglia [237,238,239,240]. Each astrocyte occupies a non-overlapping spatial domain and extends fine processes that contact thousands of synapses and neuronal soma [241,242]. This intimate association forms the so-called “tripartite synapse”, which integrates astrocytes into the functional structure of synaptic transmission and constitutes a core architecture for neuron–glia signaling in the NAc [243,244].

In line with this structural organization, glial fibrillary acidic protein (GFAP) is the most widely used marker for identifying astrocytes and plays diverse roles in cell migration, proliferation, synaptic remodeling, and blood–brain barrier maintenance [239]. GFAP has also been implicated in regulating the expression and function of glutamate transporters, a role particularly relevant in the NAc. Within this brain area, astrocytes are essential regulators of synaptic transmission and neural excitability. They influence motivational and reward-related behavior by releasing gliotransmitters and neuromodulators [245]. They maintain neurotransmitter balance through Ca^2+^-dependent uptake and release of glutamate and GABA [246,247], ensuring proper synaptic termination and recycling. In addition to glutamate clearance, astrocytes can also release small quantities of glutamate, coordinating neuronal firing and modulating excitatory/inhibitory balance via mechanisms resembling synaptic exocytosis.

Recent evidence highlights astrocyte–DA interactions in the NAc. For instance, astrocytes respond to synaptically released DA with increases in intracellular Ca^2+^, leading to ATP/adenosine release and subsequent suppression of excitatory transmission via adenosine A1 receptor activation [248]. Similarly, DA D2 receptor signaling modulates Ca^2+^ activity in midbrain astrocytes, indicating regional heterogeneity in astrocyte–DA interactions [249]. These findings underscore the importance of astrocytes in tuning dopaminergic and glutamatergic signaling within reward circuits.

Astrocytes also participate in neuroimmune regulation. They express receptors for cytokines and other immune signals, allowing them to sense and modulate inflammation [250,251]. Upon injury or stress, they undergo reactive gliosis—a hypertrophic response associated with the release of pro- and anti-inflammatory mediators, recruitment of microglia, regulation of immune cell infiltration, and elevated GFAP expression [252,253,254].

Relevant to the NAc, astrocytes contribute to extracellular homeostasis by regulating potassium and glutamate concentrations [255]. They detect synaptic activity through glutamate receptor activation [24,256], triggering Ca^2+^ transients that induce gliotransmitter release [257,258], which in turn influence neuronal excitability and network synchronization [208,258,259,260]. Astrocytes also generate Ca^2+^ waves across glial networks, coordinating activity within and across brain regions [261] and potentially activating microglia as early signals of glutamate dysregulation [185,262,263]. Within the cortico-accumbal circuit, astrocytes modulate glutamatergic plasticity by controlling glutamate tone: system Xc^−^ exchanges intracellular glutamate for extracellular cystine in a 1:1 ratio, accounting for over 50% of extracellular glutamate in the NAc core [264,265]. In parallel, astrocytic glutamate uptake via GLT-1 is crucial for synaptic fidelity and neuroprotection, as GLT-1 mediates over 90% of total brain glutamate clearance [266,267].

## 7. Glial Contributions to Stress-Induced Glutamate and Neuroimmune Dysregulation in the Nucleus Accumbens (NAc) Core: Microglia and Astrocyte Crosstalk

Increasingly, microglia are recognized as pivotal regulators of neurobiological adaptations to both stress [186,187] and drug exposure [17], as well as their interaction [268]. This convergence may represent a critical mechanism driving heightened vulnerability to addiction. While microglial activation has been investigated across multiple mesocorticolimbic regions, studies specifically addressing their role within the NAc—a central hub of the brain’s reward circuitry—remain relatively scarce.

In parallel, a growing body of evidence—outlined in the preceding sections—highlights the active involvement of both astrocytes and microglia, as well as their dynamic interactions, in regulating neuronal function following stress. A major advance has been the discovery of bidirectional astrocyte–microglia communication mediated by cytokines such as IL-1α, IL-1β, and TNF-α, which collectively shape neuronal circuitry under both physiological and pathological conditions [24].

Emerging evidence further supports a close link between maladaptive glutamatergic plasticity and neuroimmune processes in drug-experienced animals [269,270,271]. In this context, stress and glucocorticoids have been shown to sensitize neuroinflammatory responses to subsequent drug exposure, indicating that immune mechanisms may play a crucial role in mediating stress-induced vulnerability to addiction [231,272,273]. Moreover, several studies have documented a robust association between neuroinflammatory states and altered glutamate homeostasis in specific brain regions, including the NAc [24,274,275], thereby reinforcing the hypothesis that neuroimmune alterations may serve as upstream modulators of glutamatergic dysregulation in addiction.

### 7.1. Microglial Activation by Stress and Drugs: Neuroimmune Modulation in Mesolimbic Circuits and Peripheral Crosstalk

Notably, microglial cells respond rapidly to various stimuli, releasing pro-inflammatory cytokines, contributing to disrupt glutamate homeostasis, and participating in the remodeling of dendritic spine architecture [17,190,276,277,278].

Their dynamic ability to shift activation states (see Section 6.1) has attracted increasing attention, as stress exposure can lead to both immediate and long-lasting neuroimmune and structural alterations in stress-sensitive brain regions [17,27,186,187,279]. In this context, it becomes important to explore how exposure to stress leads to microglial activation and the subsequent release of pro-inflammatory mediators within stress-sensitive brain regions. Interestingly, compelling evidence for microglia’s central role in mediating the effects of chronic stress on cocaine vulnerability comes from Avalos et al. (2022) [85]. Male rats were exposed to chronic restraint stress or sham treatment and then underwent a cocaine self-administration protocol. Chronic stress led to a progressive escalation of cocaine intake over ten days, an effect that was completely prevented by daily administration of minocycline during the self-administration phase, maintaining intake at levels comparable to non-stressed controls. Notably, neither stress nor minocycline altered sucrose consumption in a separate cohort, indicating that the stress-induced enhancement was specific to cocaine-seeking behavior. At the cellular level, chronic restraint stress induced hyper-ramified microglial morphology in the NAc core of vehicle-treated animals, consistent with a primed, pro-inflammatory state. Minocycline effectively blocked this morphological activation. Molecular analyses revealed that stressed animals exhibited elevated TNF-α mRNA and protein, reduced GLT-1 expression, and showed signs of astrocyte hypoactivity, reflected by decreased GFAP immunoreactivity in the NAc core. Remarkably, all of these molecular alterations were reversed by minocycline, highlighting the tight link between microglial activation, astrocytic dysfunction, and stress-induced vulnerability to cocaine.

Similarly, chronic electric foot shock stress robustly activates microglia and increases pro-inflammatory cytokines, including TNF-α and IL-1β, in other stress-sensitive brain regions, such us the prefrontal cortex and hippocampus. These neuroimmune changes persist well beyond the cessation of stress and can be prevented by microglial inhibitors, mirroring findings observed in the NAc [280,281,282,283,284,285].

In addition, several paradigms—including restraint stress, repeated social defeat, and footshock—promote hyper-ramification, characterized by increased secondary branching without alterations in primary processes [227], along with elevated Iba-1 expression in other regions [229,280,286,287,288], consistent with observations in the NAc.

Glucocorticoids released in response to inescapable stress can prime hippocampal microglia to exhibit exaggerated inflammatory responses to subsequent challenges [232], raising the question of whether similar glucocorticoid-driven priming also occurs in other stress-sensitive regions such as the NAc. Notwithstanding the relationship between microglia activation, neuroimmune signaling and glutamatergic regulation has so far been demonstrated only in the NAc.

Exposure to addictive drugs can also profoundly affect microglial function and the release of inflammatory mediators, although the nature of these responses varies depending on the drug type, dose, and brain region [289]. In vitro studies have further demonstrated that cocaine exposure enhances the transcription of TNF-α and IL-6 in microglial cultures [290]. In vivo, ethanol has been shown to sensitize microglia, inducing a hyper-ramified activation state characterized by modest morphological changes and increased release of pro-inflammatory cytokines [269]. In cocaine models, microglia are also activated through Toll-like receptor 4 (TLR4), receptor for advanced glycation end-products (RAGE), and high-mobility group box 1 (HMGB1) signaling, as well as through NLRP3 inflammasome pathways, thereby linking psychostimulant action to neuroimmune cascades [17,291,292,293]. Consistent with these findings, psychostimulant administration increases Iba-1 expression across multiple brain regions, including the striatum [290]. Additionally, a recent study reported a long-lasting elevation of TNF-α in the NAc core following nicotine self-administration and extinction [271]. Collectively, these findings indicate that microglial morphological and functional alterations are common neuroimmune responses triggered by both stress and exposure to drugs of abuse.

All of these findings strongly support the notion that microglia are key mediators of the behavioral and neurobiological consequences of combined stress and cocaine exposure. Taken together, this body of evidence indicates that microglia play a pivotal role in the stress-induced escalation of cocaine intake, as well as in the associated cellular and molecular neuroadaptations—underscoring their critical involvement in the shared pathophysiology of stress-related disorders and SUDs.

#### Stress-Induced Recruitment of Peripheral Monocytes to the Brain: Role of Corticosterone, NMDA Receptors, and IL-6 Signaling

Complementing these findings, studies using restraint and social defeat stress models have demonstrated neuroimmune alterations, including microglial proliferation within the brain parenchyma and the recruitment of peripheral monocytes to several brains [294], although this latter effect has not been observed in the NAc. Notably, the redistribution and increased trafficking of peripheral monocytes have been associated with neuroimmune disturbances triggered by repeated stress exposure, events that have direct implications for synaptic plasticity and may promote the emergence of stress-related behavioral alterations [295]. In mice subjected to restraint stress, flow cytometry analyses based on CD11b/CD45 markers revealed microglial proliferation, attributed to the corticosterone-induced activation of NMDA receptors. Blocking corticosterone synthesis, glucocorticoid receptors, or NMDA receptors mitigated this stress-induced microglial expansion, and administration of the NMDA receptor antagonist MK-801 prevented proliferation even following exogenous corticosterone treatment in non-stressed animals [274]. These results align with earlier work from our laboratory, which demonstrated that amphetamine-induced immune sensitization—paralleling phenomena in the limbic system—was blocked by systemic MK-801 administration, suggesting that both processes are mediated by glutamate-dependent mechanisms [296]. While NMDA receptor blockade effectively prevents microglial proliferation induced by stress or psychostimulants, the effectiveness of other anti-inflammatory strategies remains to be fully established; thus far, additional studies are needed to determine whether minocycline or related compounds can effectively reverse microglial proliferation under stress and drug exposure. However, due to the non-specific actions of minocycline and the complexity of distinguishing central from peripheral immune responses, definitive conclusions regarding peripheral contributions under stress conditions are difficult to draw by using this drug [184,297].

Collectively, these observations suggest that chronic stress engages both central and peripheral immune mechanisms. Cytokines such as IL-6 play a central role in this immune-to-brain communication, acting as mediators between peripheral immune cells and the CNS. Repeated social defeat stress robustly increases the plasma IL-6 levels in mice, promoting the recruitment of proinflammatory monocytes to the brain, which adopt a primed profile and facilitate IL-1-mediated inflammatory responses [298]. Translationally, elevated IL-6 levels have been observed in cocaine users [299]. Stress-induced IL-6 can also cross the brain barrier following the loss of tight junction proteins such as claudin-5, thereby modulating brain parenchymal inflammation and behavior [300]. Importantly, although IL-6 contributes to peripheral-to-central immune signaling, microglial morphological activation in regions such as the NAc can occur through IL-6-independent mechanisms, consistent with findings from our laboratory showing that chronic stress-driven increases in cocaine intake do not alter IL-6 expression in the NAc, despite marked microglial activation [85,176]. Additional studies corroborate this region- and paradigm-specific pattern, reporting both unaltered and increased IL-6 levels in different brain regions and stress models [301,302,303,304].

### 7.2. Astrocyte Reactivity Under Stress and Drug Exposure: Glutamate and Immune Adaptations

Astrocytes respond to both acute and chronic injuries in the CNS [305,306]. In acute pathological conditions, astrocytes can become pro-inflammatory, and this state impairs glutamate clearance and promotes oxidative stress, thereby contributing to excitotoxic neuronal damage. In contrast, during chronic CNS injury or disease, astrocytes undergo reactive astrogliosis, with elevated expression of markers such as GFAP [307].

Typically, reduced GLT-1 levels are linked to reactive astrogliosis in response to brain injury, ischemia, or neurodegeneration, suggesting that astrocytes might adopt a reactive phenotype following drug exposure. In support of this notion, increased astrocyte reactivity has been reported after non-contingent exposure to psychostimulants such as cocaine [308]. This reactive state is often marked by elevated expression of GFAP. In vitro studies demonstrated that methamphetamine induces robust activation of purified cortical astrocytes via a protein kinase C (PKC)-dependent mechanism. Furthermore, sustained astrocytic activation was observed in cortical neuron–glia cocultures following methamphetamine exposure [309,310]. These in vitro findings are consistent with in vivo evidence showing that the repeated administration of methamphetamine leads to behavioral sensitization, which is accompanied by PKC-dependent astrocytic activation in the cingulate cortex and NAc. Similarly, morphine also induced astrocytic activation through PKC signaling in cortical neuron–glia cocultures, although purified astrocytes did not respond directly to morphine [311].

Despite evidence of astrocyte activation during experimenter-administered drug exposure, cocaine self-administration followed by extinction training leads to astrocytes in the NAc exhibiting reduced GFAP expression, decreased surface area and volume, and diminished colocalization with the presynaptic marker synapsin I [312], while no such changes occur in the prelimbic cortex or basolateral amygdala [313]. Importantly, astrocyte morphology and synaptic proximity remain unchanged after self-administration alone, with alterations emerging only after extinction/abstinence, underscoring the region-specific and time-dependent nature of astrocyte adaptations to cocaine exposure. Similarly, following methamphetamine self-administration and extinction, astrocytes in the NAc core show reduced contact of their perisynaptic processes (PAPs) with synapses, indicative of astrocytic process retraction [314], although this morphological change does not impair GLT-1 expression or glutamate uptake capacity. Together, these findings emphasize the complexity of psychostimulant-induced neuroadaptations and underscore the contribution of astrocytic dysfunction to synaptic alterations that may underlie relapse vulnerability.

Consistent with this evidence, this population of glial cells does not exhibit a reactive phenotype under stress conditions, proving on the contrary a hypo-reactive state [85,315]. For example, early or juvenile stress has been shown to adversely affect astrocytic function, leading to diminished GFAP expression, less complex astrocyte morphology, and impaired uptake of key neurotransmitters such as glutamate and GABA. Additionally, disruptions in the glutamate–glutamine cycle have been reported under these conditions [316]. Experimental models of early life stress, such as repeated maternal separation or deprivation during lactation, consistently show marked reductions in GFAP-immunoreactive astrocytes across multiple brain regions [301,317,318]. Although a consistency in GFAP reduction and astrocyte complexity across different brain regions following stress has been shown, recent evidence from chronic stress and systemic inflammation paradigms reveals a more complex picture. Using the unpredictable chronic mild stress model and LPS injections, astrocyte activation increased—evidenced by higher GFAP fluorescence, greater branch bifurcation, and enhanced arborization—in multiple brain regions, including the NAc, with females showing more pronounced effects in the hippocampus and amygdala than males [319]. These findings highlight the region- and sex-specific nature of astrocyte responses to diverse stress paradigms and inflammatory stimuli (i.e., LPS), potentially contributing to differential vulnerability to stress-related disorders. Consistent with this idea, our laboratory demonstrated that chronic restraint stress, which underlies stress-induced cocaine self-administration, is associated with reduced astrocyte reactivity in the NAc core evidenced by decreased GFAP immunoreactivity [85].

### 7.3. Microglia–Astrocyte Crosstalk: Proinflammatory Signaling, GLT-1 Downregulation and Stress-Induced Vulnerability to Cocaine Use Disorder

Microglia–astrocyte crosstalk plays a central role in the neuroadaptations induced by chronic stress that enhance vulnerability to cocaine addiction. Stress induces profound structural and functional changes in both astrocytes and microglia—two glial populations essential for glutamatergic regulation and synaptic homeostasis—particularly in the NAc core [84,85,130].

Elevated extracellular glutamate induced by chronic stress serves as an alarm signal that drives microglial activation through NMDA receptor-dependent mechanisms, particularly in the context of corticosterone release [85,130,143,274,320]. Once activated, microglia release pro-inflammatory cytokines such as TNF-α that contribute to the disruption of glutamate homeostasis by modulating astrocytic function [321,322,323,324,325,326] and postsynaptic outcomes [27]. Although astrocytes can produce TNF-α at low levels, transcriptomic analyses consistently identify microglia as the primary CNS source [327,328,329,330,331,332]. Stress and drugs of abuse elevate TNF-α, which act at glial and postsynaptic levels to perpetuate glutamatergic dysregulation [24,27,271,333,334]. Microglia thus appear to occupy an upstream position in regulating glutamate uptake and excitatory transmission, while astrocytes can reciprocally influence microglial activation through the release of ATP, glutamate, and other modulatory signals, supporting bidirectional crosstalk [335,336]. Mechanistically, TNF-α downregulates astrocytic GLT-1 expression and impairs its functional activity through transcriptional repression [211,213,337,338,339,340,341], consistent with a glutamate-to-cytokine feed-forward loop, as restraint stress increases cortical TNF-α in a glutamate-dependent manner, an effect blunted by NMDA receptor antagonism [342]. IL-1β may likewise reduce GLT-1 through post-translational or GFAP-independent mechanisms [343], although in our studies, chronic stress capable of impairing glutamate homeostasis and facilitating cocaine self-administration does not necessarily increase IL-1β expression in the NAc [85].

Chronic stress not only produces a sustained decrease in GLT-1, but also reduces astrocytic GFAP immunoreactivity in the NAc core [85,344]. Because GFAP provides structural support for GLT-1 membrane trafficking [345], and neuron–astrocyte communication further governs GLT-1 transcription and membrane dynamics [346,347,348], such alterations in glutamatergic signaling critically impair astrocytic function and extracellular glutamate control.

Importantly, microglial hyperactivation appears to precede and drive astrocytic dysfunction, positioning microglia upstream in the control of glutamate uptake and excitatory transmission [200,233]. In summary, chronic stress disrupts the finely tuned interactions among astrocytes, microglia, and neurons in the NAc core, resulting in impaired glutamate clearance and its extracellular accumulation. In this context, TNF-α signaling has emerged as a key factor, being implicated both in GLT-1 downregulation [211,212,213,339] and in the regulation of synaptic plasticity [209,349]. Despite significant advances, the specific microglial mechanisms governing glutamate homeostasis within the NAc core under conditions of stress–drug comorbidity remain to be fully elucidated.

#### TNF-α/NF-κB Pathway: A Key Signaling Axis Driving Astrocyte–Microglia Crosstalk in Stress-Induced Cocaine Vulnerability

As discussed above, and consistent with the general alterations in cytokine levels induced by stress and drugs, elevated TNF-α levels have been reported in several rodent models of chronic stress and in individuals with mood disorders [350,351], highlighting its role as a key mediator in astrocyte–microglia communication. Importantly, the transcription factor NF-κB acts as a pivotal regulator of this inflammatory response by controlling the transcription of TNF-α. Through its activation, NF-κB mediates stress-induced synaptic plasticity changes driven by cytokine signaling, thus linking neuroinflammation to behavioral adaptations [352,353].

**Figure 3:**
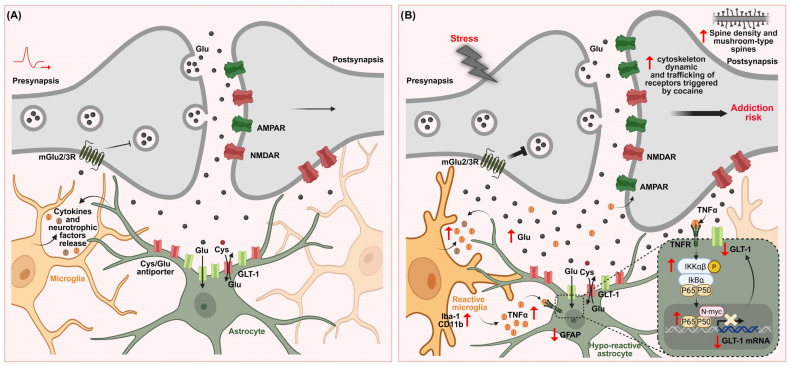
Microglia–Astrocyte Crosstalk in Stress-Induced Vulnerability to Cocaine Use Disorder. Panel (**A**) depicts a tripartite synapse within the NAc core under control conditions, illustrating the normal interactions among presynaptic terminals, postsynaptic medium spiny neurons (MSNs), and perisynaptic astrocytic processes that collectively maintain glutamate homeostasis in the region. Panel (**B**) illustrates the cascade of events initiated by chronic stress, which elevates extracellular glutamate in the NAc core and activates microglia through ionotropic and metabotropic receptors. Activated microglia undergo hyper-ramification and release proinflammatory cytokines, primarily TNF-α. This cytokine promotes the nuclear recruitment of NF-κB, resulting in transcriptional repression of astrocytic GLT-1. TNF-α also diminishes GFAP-associated structural integrity, further compromising glutamate clearance. The resulting glutamate spillover drives extrasynaptic NMDA/mGluR activation, leading to postsynaptic remodeling of MSNs, characterized by increased mushroom-type dendritic spines and increased AMPA receptor trafficking. These adaptations heighten vulnerability to cocaine reinforcement and relapse-like behaviors, which can be prevented by pharmacological restoration of microglial homeostasis with minocycline. This figure was created by BioRender.com.

The signaling pathway involving TNF-α/NF-κB has been widely investigated in the brain under both normal and disease conditions [352,354]. In the CNS, TNF-α commonly initiates the canonical NF-κB pathway, which depends on the phosphorylation of IKKα/β subunits. This event promotes the degradation of IκBα, allowing NF-κB heterodimers to translocate into the nucleus [355]. Within the nucleus, NF-κB interacts with κB elements in the DNA to modulate the transcription of numerous genes, particularly those related to inflammatory processes and glutamate signaling [356,357]. Because of the influence of NF-κB-regulated genes on drug-seeking behavior, it has been identified as a crucial mediator of the neuroadaptations triggered by chronic drug exposure [358]. Chronic cocaine exposition increases both the total protein levels of the p50/p65 NF-κB subunits and overall NF-κB activity in the NAc [359,360]. Reducing NF-κB activity in this region diminishes cocaine reward and blocks the increase in dendritic spine density typically observed after prolonged exposure [360]. Inhibition of NF-κB signaling in the NAc core also reduces cue-induced cocaine seeking in a sex-dependent manner [361]. Recent evidence demonstrates robust activation of the cortical microglial TLR4/NF-κB signaling pathway after methamphetamine self-administration. Inhibition of this pathway effectively attenuates neuroinflammation and reduces addiction-related behaviors [362]. Stress, on the other hand, is also a well-established activator of NF-κB. Persistent NF-κB activation has been reported in the hippocampus following predator scent stress, and in the prefrontal cortex after exposure to immobilization/acoustic stress [363,364]. Manipulation of NF-κB signaling in the ventral striatum via viral vectors leads to a reduction in behavioral sensitivity to chronic social defeat stress, underscoring the involvement of IKK in the neuroplastic changes driven by stress [365]. In line with these findings, recent data from our lab demonstrated a marked activation of NF-κB in the NAc core from chronic stressed rats. Consistently, viral NF-κB inhibition prevented stress-induced facilitation of cocaine self-administration and cross sensitization [176]. The impact of viral NF-κB inhibition on stress-induced enhancement of cocaine intake is, at least in part, mediated by the restoration of GLT-1 levels within the NAc, leading to the normalization of glutamate homeostasis. In this sense, these results notably support that NF-κB regulates the expression of astrocytic GLT-1 [340]. TNF-α is typically recognized as an activator of NF-κB-dependent gene expression; however, it can also promote NF-κB-mediated transcriptional repression, as observed by the TNF-α/NF-κB-induced downregulation of GLT-1—a process in which N-myc proto-oncogene protein (N-myc) is involved [339]. Specifically, in the context of motor neuron injury, microglial TNF-α has been shown to downregulate astrocytic GLT-1 through the NF-κB pathway, enhancing glutamate-mediated excitotoxicity and contributing to motor neuron death in amyotrophic lateral sclerosis [213]. Under conditions of stress and drug exposure, TNF-α/NF-κB signaling plays a critical role in chronic stress-induced GLT-1 downregulation within the NAc core and the increased escalation of drug self-administration of cocaine self-administration, with N-myc appearing to contribute to NF-κB-mediated transcriptional repression [176]. These findings are consistent with those reported by Namba et al., 2022 [361] following cocaine exposure. We further showed that stress-induced NF-κB signaling occurs specifically in astrocytes within the NAc core. Interestingly, the NF-κB transcriptomic profile did not correlate with GLT-1 expression changes in the prefrontal cortex following extinction training after cocaine self-administration [366], suggesting that NF-κB may exert region-specific regulatory control over GLT-1 expression under conditions of drug exposure. Notwithstanding, these results support a pivotal role for TNF-α/NF-κB signaling in mediating astrocyte–microglia crosstalk, potentially driving the neuroplastic changes associated with stress-related vulnerability to cocaine addiction (see Table 1 and Figure 3 for key finding from Section 5 and Section 7).

## 8. Microglia–Astrocyte Crosstalk in the Regulation of Structural Synaptic Plasticity in the Nucleus Accumbens (NAc) Core and Its Role in Stress-Induced Cocaine Vulnerability

Stress-induced plasticity shifts are often maladaptive, contributing to craving, depressive-like behaviors, cross-sensitization to psychostimulants, and the escalation of drug self-administration [367,368,369,370]. Particular emphasis is placed on immune-regulatory processes primarily mediated by microglia, including the release of proinflammatory cytokines such as TNF-α and IL-1β, which critically modulate synaptic strength and structural plasticity within reward-related circuits. These microglial-derived signals influence astrocytic glutamate transporters and receptor dynamics, thereby linking stress-induced neuroimmune activation to maladaptive synaptic remodeling and, ultimately, to heightened vulnerability to SUDs.

Microglia—beyond their classical immune surveillance function—have emerged as key regulators of neuroplasticity [269,334,371,372,373]. Early developmental studies first demonstrated their essential role in sculpting synaptic plasticity [189]; however, their precise role in modulating synaptic circuits in the adult brain remains incompletely understood [185,188,194,374].

Chronic stress has been shown to alter both microglial morphology and functionality [286,288], and these microglial alterations often coincide with neuronal structural and functional changes that may underlie long-lasting emotional disturbances associated with mood disorders such as major depression [375]. Consistently, Gaspar et al. (2021) [376] reported that unpredictable chronic mild stress induces morphological modifications in microglia and neurons within the NAc, with distinct sex-dependent features. More recent studies indicate that microglia actively shape neuronal networks by directly interacting with neurons or by releasing bioactive molecules such as cytokines—a mechanism susceptible to disruption by both stress and addictive drugs [268,377]. In line with this, inhibition of stress-induced microglial remodeling prevents the associated increases in NAc spine density and excitability in cocaine-exposed mice [378]. Consistently, evidence from our laboratory [85,176] supports a link between stress-induced microglial activation and structural plasticity that ultimately modulates cocaine-induced behavioral responses.

Delving into the features of both stress- and drug-induced alterations in synaptic plasticity, it is important to emphasize that the glutamatergic postsynapse represents a critical convergence point for mechanisms underlying stress- and addiction-related adaptations in MSNs of the NAc. Dendritic spine remodeling—reflected in the balance between mature and immature spines—serves as a structural correlate of experience-dependent synaptic strength [21,379,380,381]. Drugs of abuse, such as cocaine, robustly increase dendritic spine density in NAc MSNs [382,383,384,385,386]. These structural changes are reflected in enhanced actin cytoskeleton remodeling, increased surface expression of AMPA (GluR1) receptors, and elevated AMPA/NMDA receptor ratios—synaptic adaptations that underlie potentiated excitatory transmission [21,169,387,388,389]. Following prolonged abstinence, re-exposure to psychostimulants or drug-associated cues induces a rapid and transient enlargement of dendritic spine heads in NAc MSNs, accompanied by dynamic changes in actin polymerization and AMPA receptor trafficking; these effects typically normalize within approximately two hours [390,391,392].

Similarly, stress induces convergent structural adaptations within the NAc core, including increased densities of total and mushroom-type spines, with specific patterns depending on the stress paradigm, MSN subtype, and timing of assessment [84,365,393,394]. Larger “mushroom” spines form stronger excitatory synapses [395] and are associated with enhanced AMPA receptor insertion and remodeling of the postsynaptic density [396,397,398,399,400,401]. Chronic stress also elevates basal extracellular glutamate levels [143,320,344] and promotes cytoskeletal remodeling, possibly as a homeostatic compensatory adaptation [21,402]. Within this framework, stress may “prime” the NAc core by expanding the pool of structurally mature spines, thereby facilitating subsequent cocaine-induced synaptic potentiation. This primed state may enable cocaine exposure to transform latent structural potential into functional plasticity through AMPA receptor trafficking and insertion into the postsynaptic membrane [84,129,130,152,369].

Microglial sensing of glutamate can drive cytokine-mediated forms of synaptic plasticity. Among these, TNF-α and IL-1β exert modulatory effects on glutamate homeostasis both directly at the postsynaptic level and indirectly through glial-mediated mechanisms involving microglia–astrocyte crosstalk. Concerning their direct actions on postsynaptic function, these pro-inflammatory cytokines influence long-term potentiation (LTP) and synaptic scaling. TNF-α, for instance, enhances synaptic efficacy by increasing surface AMPA receptor expression, whereas blockade of TNF-α signaling reduces synaptic strength [403]. Moreover, TNF-α mediates homeostatic synaptic scaling, adjusting synaptic weights to stabilize network activity following prolonged activity blockade [209]. Nonetheless, TNF-α appears to be dispensable for acute forms of plasticity, as most studies report no significant effect on LTP after TNF-α manipulation [210,404].

Elevated IL-1β levels, in turn, affect both the induction and maintenance phases of LTP [405,406]. Importantly, IL-1β can disrupt neuronal function and synaptic plasticity by interfering with both LTP and long-term depression (LTD), two complementary mechanisms underlying learning and memory [407,408]. Mechanistically, IL-1β selectively regulates AMPA receptor phosphorylation and surface expression through extracellular calcium- and NMDA receptor-dependent signaling in hippocampal neurons [409]. Additionally, IL-1β induces the loss of PSD-95—a scaffold protein critical for synapse maturation and stability—in neuronal cultures [410]. Altogether, these findings underscore the central role of inflammatory cytokines in regulating neuronal plasticity, bridging adaptive homeostatic processes with maladaptive neuroinflammatory responses.

Crucially, Avalos et al. (2022) [85] demonstrated that chronic restraint stress is associated with increased NAc core microglial hyper-ramification and elevated mushroom-type spine density, along with higher TNF-α (mRNA/protein) levels and decreased GLT-1 and GFAP expression—changes consistent with astrocytic hypoactivity. Daily minocycline administration prevented all of these adaptations, indicating a microglia-dependent cascade linking stress to glutamatergic dysregulation and structural remodeling, ultimately facilitating cocaine self-administration.

Collectively, these findings support a mechanistic framework in which chronic stress engages microglia–astrocyte interactions to regulate and sense extracellular glutamate while promoting the release of proinflammatory cytokines—particularly TNF-α and IL-1β. Through these inflammatory and non-inflammatory pathways, microglia contribute to both homeostatic and maladaptive forms of plasticity that drive stress-induced vulnerability to drug addiction (see Table 2 and Figure 3 for key finding from this Section).

## 9. Repurposing Glutamatergic Therapies for the Treatment of Substance Use Disorders (SUDs) Comorbidity

In recent years, research on treatments for stress and SUDs has grown substantially. Current initiatives continue to explore and assess innovative therapeutic options, including both psychological interventions and pharmacological strategies. There is increasing interest in discovering pharmacological treatments that can reduce the symptoms associated with PTSD and SUDs. Regarding PTSD, the FDA has authorized two selective serotonin reuptake inhibitors—paroxetine and sertraline—for clinical use. To date, no pharmacological treatment has been approved for cocaine addiction, or for the comorbid presentation of PTSD, despite the high clinical need. Efforts to develop effective pharmacotherapies for this comorbidity have focused on evaluating compounds that target shared neurobiological mechanisms underlying both disorders. Thus, in recent years, the repurposing of drugs that modulate glutamatergic transmission has emerged as a promising therapeutic avenue for treating the comorbidity between cocaine addiction and stress-related disorders. In the following section, we discuss how this approach has expanded to include pharmacotherapies aimed at modulating glial components as a potential strategy for addressing comorbid PTSD and SUDs, with a particular focus on cocaine.

### 9.1. N-Acetylcysteine (NAC)

N-acetylcysteine (chemical formula C_5_H_9_NO_3_S) (NAC) is a cysteine prodrug. For more than 30 years, NAC has been used clinically as an antidote for acetaminophen poisoning [411].

NAC exerts its effects in the brain through various mechanisms. As an acetylated derivative of cysteine, it has good bioavailability and can cross the blood–brain barrier. Cystine acts as the substrate for the system Xc^−^, which facilitates the import of cystine into the cell in exchange for glutamate. Through this exchange, the transporter plays a key role in regulating extracellular glutamate levels. Once internalized, cystine is reduced to cysteine, the rate-limiting precursor for the synthesis of glutathione (GSH), a crucial endogenous antioxidant [412].

Due to its action on system Xc^−^, capable of influencing extracellular glutamate levels, NAC has shown potential in regulating not only the glutamatergic system but also dopaminergic transmission. Thus, NAC treatment has been consistently investigated in preclinical studies as a potential therapy for cocaine dependence, and its evaluation has also extended to promising clinical trials.

In preclinical models, pretreatment with NAC (60 mg/kg i.p.)—administered after each self-administration session or prior to each injection during a 7-day non-contingent cocaine regimen—effectively prevented cocaine-induced escalation of drug intake, behavioral sensitization, and drug-primed reinstatement [413]. Importantly, NAC (30–90 mg/kg) reduced cocaine seeking in a dose-dependent manner during both early and late addiction stages, without affecting cocaine reinforcement, supporting its potential for relapse prevention [414]. However, when administered after the establishment of escalated cocaine intake, chronic NAC treatment (60 mg/kg) had no measurable effect on further escalation or on motivation for cocaine under a progressive ratio schedule. Instead, NAC promoted abstinence by increasing sensitivity to punishment, suggesting a selective role in restoring behavioral control in the face of adverse consequences [415]. Consistent with these behavioral findings, NAC restores glutamate homeostasis by enhancing the activity of the cystine–glutamate exchanger, thereby normalizing extracellular glutamate levels in animals with a history of drug exposure [159]. Moreover, administration of NAC (100 mg/kg i.p.) for five consecutive days prior to reinstatement testing attenuated both stress-enhanced alcohol and cocaine consumption, as well as the reinstatement of alcohol- and cocaine-seeking behaviors triggered by conditioned stress cues in rats [416].

Mechanistically, stimulation of mGluR2/3 receptors by non-synaptic glutamate derived from the cystine–glutamate exchanger inhibits synaptic glutamate release and, consequently, excitatory synaptic activity, an effect believed to underlie the ability of NAC to suppress cocaine-associated behaviors [159]. Accordingly, NAC treatment prevented the persistent reduction in system Xc^−^ activity, preserving cystine transport, basal extracellular glutamate levels, and cocaine-evoked glutamate release in the NAc [417]. NAC (33 mg/kg i.p., administered 20 min prior to a cocaine priming injection) also restored synaptic plasticity in the NAc of cocaine-exposed rats by stimulating mGluR2/3 and mGluR5, reversing cocaine-induced metaplasticity and supporting its anti-relapse effects [418]. Consistently, administration of the mGluR2/3 antagonist LY341495 blocked the inhibitory effect of NAC on reinstatement in animals trained to self-administer cocaine [419]. In addition to its action on cystine–glutamate exchange, evidence suggests that the restoration of GLT-1 may also constitute a key mechanism by which repeated NAC treatment reduces cue-induced cocaine reinstatement [171].

Several clinical studies have explored the effects of NAC on cocaine use, with mixed results. Early short-term, placebo-controlled trials in hospitalized, non-treatment-seeking cocaine-dependent individuals found that NAC (1200 mg/day) reduced cue-reactivity and showed trends toward decreased craving and withdrawal symptoms, although some effects were not statistically significant, likely due to small sample sizes [420,421]. Open-label studies further demonstrated that NAC was well tolerated at doses up to 3600 mg/day and associated with reduced self-reported cocaine use and craving [422,423]. However, a larger double-blind placebo-controlled trial in 111 treatment-seeking adults did not find significant effects of NAC on abstinence, although subgroup analysis revealed that NAC increased time to relapse and reduced craving in already abstinent individuals [424], supporting the idea that NAC may be more effective as a relapse prevention strategy rather than a treatment for ongoing use. Recently, a randomized, placebo-controlled crossover trial demonstrated that short-term NAC administration (2400 mg/day for 2 days) significantly reduced cocaine cue-related reactivity in individuals with cocaine use disorder, without changes in subjective craving [425].

In line with findings from preclinical research, a double-blind, 8-week clinical trial was conducted in veterans (*n* = 35) diagnosed with PTSD and SUDs, who received either NAC (2.4 g/day) or placebo in combination with cognitive-behavioral therapy for SUDs. NAC treatment led to reductions in PTSD symptoms, as reported by both the participants and clinicians, as well as in drug craving. Notably, these improvements persisted for at least one month after discontinuation of the pharmacological treatment [426].

As another relevant mechanism, NAC may play a role in modulating immune responses. NAC modulates the expression and secretion of inflammatory cytokines under various inflammatory conditions both in vitro and in vivo. As a precursor of GSH and direct antioxidant, NAC inhibits NF-κB activation and reduces the expression of pro-inflammatory genes. It significantly suppresses IL-1β release induced by lipopolysaccharides. Additionally, NAC reverses cytokine alterations triggered by social isolation, including elevated IL-6 and IL-4 and reduced TNF-α and IFN-γ levels [427,428,429]. As extensively discussed in this review, increased levels of inflammatory mediators—such as TNF-α/ NF-κB signaling—have been reported in individuals with comorbid stress and SUDs. Considering that both neuroinflammation and stress-induced adaptations at glutamatergic synapses play a key role in the pathophysiology of PTSD and SUDs [430], NAC may hold therapeutic potential for treating this comorbid condition, potentially also by modulating its neuroinflammatory component.

### 9.2. Ceftriaxone

Ceftriaxone is a third-generation cephalosporin antibiotic known not only for its antimicrobial properties but also for its ability to modulate the glutamatergic system within the CNS.

Rothstein et al., 2005 [431] was the first to identify β-lactam antibiotics as potent inducers of GLT-1 expression, primarily by promoting its gene transcription. In this study, ceftriaxone-induced upregulation of astrocyte GLT-1 expression and functional activity conferred neuroprotective effects in both in vitro and in vivo models of ischemia and amyotrophic lateral sclerosis (ALS), two conditions characterized by glutamate-induced toxicity.

Given the capacity of ceftriaxone to restore GLT-1 levels in the NAc, this treatment resulted in a reduction in drug-seeking behavior in animals with a history of cocaine intake [160]. Specifically, ceftriaxone treatment (200 mg/kg/day for 7 consecutive days), administered following each extinction session, attenuates the reinstatement of cocaine-seeking behavior induced by either cocaine-associated cues or the drug. The ability of ceftriaxone to modulate reinstatement behavior and GLT-1 expression after cocaine exposure is dose-dependent. Doses of 100 and 200 mg/kg effectively elevate GLT-1 levels and reduce cue-induced reinstatement, whereas a lower dose of 50 mg/kg given over 5 days fails to produce these effects [432]. Supporting this, glutamate uptake studies have shown that administering 200 mg/kg of ceftriaxone for 5 days restores both GLT-1 and also system Xc^−^ activity in the NAc core following cocaine self-administration [161]. This restoration leads to elevated basal extracellular glutamate and a reduction in glutamate release during cocaine-primed reinstatement. The enhancement of glutamate clearance prevents extracellular glutamate overflow, thereby attenuating drug-induced neuroplasticity and behavioral sensitization [21].

Interestingly, although ceftriaxone significantly reduced reinstatement even weeks after treatment had ended, its administration for 5 days prior to the initiation of cocaine self-administration did not affect the acquisition of drug-taking behavior. Moreover, ceftriaxone attenuated the locomotor response to the first cocaine injection and prevented the development of cocaine sensitization [433]. These findings extend to behavioral studies, in which chronic ceftriaxone administration has been shown to attenuate psychomotor sensitization induced by amphetamine [434]. Ceftriaxone reduces cocaine-seeking in rats after 21 to 45 days of abstinence without extinction, increasing GLT-1 expression in both the NAc core and shell [435,436]. However, only GLT-1 in the core is necessary to attenuate cocaine relapse [436]. At doses of 100 and 200 mg/kg, ceftriaxone decreases context-induced relapse, but only 200 mg/kg elevates GLT-1 in the core [435]. Thus, GLT-1 regulation in the core is critical for its protective effects in the absence of extinction. In line with these preclinical data, findings from our lab demonstrated that ceftriaxone restores NAc core GLT-1 expression and function and reduces stress-induced locomotion in rats exposed to acute and chronic immobilization stress, although it does not reverse the stress induced increases in AMPA/NMDA ratio [84,151]. Importantly, the restoration of glutamate transport in the NAc core by ceftriaxone prevented the capacity of acute stress to augment the acquisition of cocaine self-administration [84]. Mechanistically, the upregulation of GLT-1 expression by ceftriaxone is mediated through the canonical NF-κB transcriptional pathway [437]. Consistent with this mechanism, data from our laboratory indicate that genetic inhibition of NF-κB activity in the NAc core is sufficient to restore GLT-1 levels reduced by chronic stress exposure and prevent the stress-induced facilitation of cocaine self-administration [176]. Despite its preclinical efficacy, ceftriaxone has not yet been clinically evaluated for the treatment of stress and comorbid SUDs in a consistent way.

### 9.3. Minocycline

Minocycline is a second-generation tetracycline antibiotic and has been explored as a potential treatment for a wide range of CNS disorders. Minocycline confers neuroprotection primarily through the inhibition of microglial activation through multiple mechanisms [438], including the suppression of the pro-inflammatory NF-κB intracellular signaling pathway [439]. It has been shown, for instance, to markedly reduce LPS-induced microglial activation—as indicated by Iba1 expression—and to decrease the release of pro-inflammatory cytokines such as IL-6 [440,441], TNF-α [442], and chemokines like CCL2 [443]. Moreover, minocycline engages additional molecular targets, further contributing to its broad therapeutic potential. For example, minocycline inhibits matrix metalloproteinases (MMPs), particularly MMP-12, which are involved in blood–brain barrier breakdown and neuroinflammation [444].

Based on its pharmacological profile, several studies have evaluated its therapeutic potential in the context of psychostimulant use disorders. Thus, minocycline (40 mg/kg) reduced hyperlocomotion in mice following a single methamphetamine administration (3 mg/kg) and attenuated the development of behavioral sensitization induced by repeated exposure to the drug (3 mg/kg/day for 5 days) [445]. This effect appears to be dose-dependent, as lower doses (10 and 20 mg/kg) were ineffective. Importantly, the effective 40 mg/kg dose did not produce locomotor effects on its own, indicating that the attenuation was not due to general sedation or motor impairment. In line with this report, a previous study showed that a high dose of minocycline (100 mg/kg s.c.) reduced the locomotor-stimulating effects of a moderate dose of amphetamine (0.5 mg/kg i.p.) in rats [446]. Similarly, minocycline has been shown to suppress methamphetamine-induced glial activation and reduce intravenous methamphetamine self-administration in Long–Evans rats. When administered intraperitoneally once daily at doses of 10, 30, or 60 mg/kg for three consecutive days during self-administration sessions, minocycline significantly decreased responding maintained by the 0.03 mg/kg/infusion methamphetamine dose under a fixed ratio 1 (FR1) schedule [372].

Minocycline interferes with the development of cocaine-induced locomotor sensitization but not with its expression in male C57BL/6J mice. A high dose (80 mg/kg) reduced locomotor activity both when given alone and when administered 30 min before cocaine. Conversely, a lower dose (40 mg/kg), administered 3 h prior to cocaine, prevented the progressive increase in locomotor activity typically observed after repeated cocaine exposure, without affecting the acute locomotor response [447].

The inhibition of microglia reactivity by minocycline treatment (40 mg/kg/day) administered three days prior to stress exposure significantly alleviated PTSD-related anxiety and contextual fear in mice [448]. Similarly, minocycline (35 mg/kg) attenuated anxious-like behaviors in stress-exposed rats and stress-induced elevations of pro-inflammatory cytokines (IL-1, IL-6, TNF-α) in stress-sensitive brain areas. Consistent with these findings, our laboratory demonstrated that minocycline (30 mg/kg i.p., every 12 h for 5 days), when administered 16 days after the initial stress episode, prevents chronic restraint stress-induced escalation of cocaine self-administration [85].

Clinical research on minocycline for PTSD and anxiety related-disorders, with or without comorbid depression, is still limited but promising. In a small open-label pilot study, minocycline (100–200 mg/day) reduced PTSD symptoms and lowered CRP levels, although IL-6 and TNF-α responses were variable, suggesting complex inflammatory kinetics [449]. In patients with comorbid anxiety and depression, minocycline showed a trend toward reducing anxiety symptoms without significantly affecting depression scores in individuals primarily diagnosed with Major Depressive Disorder, with effects persisting post-treatment [450]. Another randomized controlled trial supported anxiolytic and antidepressant benefits in generalized anxiety disorder [451], though other trials in treatment-resistant depression showed no overall effect unless stratified by inflammation levels, which revealed improved antidepressant but not anxiolytic responses [452].

While these preclinical and clinical findings support the therapeutic promise of minocycline in modulating neuroinflammation and glutamatergic imbalance associated with stress-related SUDs, its clinical efficacy in addressing addiction and stress comorbidity remains to be determined.

### 9.4. Ampicillin/Sulbactam (AMP/SUL)

Sulbactam is a β-lactamase inhibitor that lacks intrinsic antibacterial activity when used alone. Recent studies have demonstrated that sulbactam can upregulate GLT-1 expression and enhance glutamate uptake, thereby exerting neuroprotective effects in models of cerebral ischemia [453,454] and Alzheimer’s disease [455].

On the other hand, ampicillin, a β-lactam antibiotic previously identified as a GLT-1 upregulator [431], has also been evaluated in preclinical studies for its therapeutic potential in various SUDs, particularly alcohol consumption [456,457]. Specifically, ampicillin (100 mg/kg i.p.), administered for 5 consecutive days to rats with high ethanol intake, effectively reduced alcohol consumption and restored GLT-1 levels in the NAc and PFC. This effect was mediated through AKT phosphorylation, a signaling mechanism previously implicated in the upregulation of GLT-1 [456]. Notably, ampicillin also increased the expression of xCT and both GLT-1 isoforms (GLT-1a and GLT-1b) in the NAc, further supporting its potential to modulate glutamate homeostasis and attenuate ethanol consumption [457]. The combination of ampicillin and sulbactam (AMP/SUL) exerts synergistic effects by enhancing glutamate transporter regulation and reducing neuroinflammation [458]. Thus, AMP/SUL (200 mg/kg i.p.) reduced cocaine-induced reinstatement [459]. These effects were linked to restoration of GLT-1 and xCT in the NAc core and shell, together with mGluR1 upregulation in the NAc. Similar results were observed in a model examining the facilitatory effect of cocaine on ethanol relapse-like behavior in male alcohol-preferring rats [460], further highlighting the effect of AMP/SUL on glutamatergic signaling during cocaine and ethanol co-exposure [460].

To date, no clinical trials in humans have evaluated the use of AMP/SUL for the treatment of comorbidity between stress and SUDs, or for any specific SUDs. The evidence available regarding AMP/SUL in this context is limited to preclinical research. Moreover, its effects have not been assessed in preclinical models of comorbidity, nor in models of stress or post-traumatic stress. Nevertheless, its potential to modulate glutamatergic signaling in brain reward areas makes it a promising target for further investigation as a therapeutic approach for these disorders (see Table 3 for key finding from this Section).

## 10. Conclusions

Microglia–astrocyte interaction emerges as a pivotal cellular substrate linking stress and addiction through both inflammatory and non-inflammatory mechanisms. Stressors and drugs of abuse consistently induce microglial activation, triggering pro-inflammatory cytokine release, glutamate dysregulation, and dendritic spine remodeling. These effects converge in the NAc core, where microglial sensitivity to extracellular glutamate appears particularly important for amplifying stress-induced neuroadaptations that favor drug seeking. Importantly, chronic stress primes microglia into a hyper-ramified state, sensitizing them to subsequent cocaine exposure and promoting exaggerated inflammatory and synaptic responses—a mechanism that may underlie heightened addiction vulnerability following prior stress.

In parallel, addictive substances directly engage microglial–astrocyte communication. Cocaine, ethanol, nicotine, and psychostimulants induce morphological and inflammatory changes across reward-related brain regions, highlighting microglia as common effectors of drug-induced neuroadaptations. Notably, studies combining chronic stress with cocaine self-administration demonstrate that microglia mediate the escalation of intake and associated molecular and structural plasticity. Pharmacological inhibition with minocycline reverses these alterations, reinforcing the translational potential of targeting microglial function.

Beyond inflammatory effects, microglia also regulate neuronal plasticity through non-inflammatory mechanisms such as synaptic pruning, gliotransmission, and trophic support. These functions are particularly relevant as maladaptive synaptic plasticity represents a shared substrate for both stress and SUDs. Taken together, microglia occupy a central position at the intersection of stress and addiction neurobiology, and future studies should clarify the balance between their inflammatory and non-inflammatory roles, regional specificity, and the contribution of peripheral immune cells.

### 10.1. Mechanistic Synthesis

- Stress-related vulnerability to cocaine use disorders arises from the interplay of HPA axis activation, neuroimmune signaling, and glial dysfunction.
- Microglial TNF-α release and subsequent downregulation of astrocytic GLT-1 emerge as central pathways linking stress to glutamatergic dysregulation in the NAc core.
- Glial crosstalk between microglia and astrocytes critically shapes synaptic plasticity, structural remodeling, and ultimately behavioral vulnerability to cocaine.

### 10.2. Weaknesses and Knowledge Gaps

- Most preclinical evidence derives from male rodents; studies in females are limited despite evidence that gonadal hormones (e.g., estrogens) modulate neurobiological processes underlying stress and drug responses.
- Clinical validation of glutamate-modulating drugs (minocycline, ceftriaxone, NAC, AMP/SUL) remains scarce, leaving a translational gap between promising preclinical findings and patient applications.
- Integration of mechanistic rodent data with human neuroimaging and biomarker studies remains insufficient.

### 10.3. Future Directions

- Mechanistic studies dissecting inflammatory versus non-inflammatory microglial functions, as well as peripheral–central immune crosstalk, in the comorbidity between stress and SUDs.
- Systematic inclusion of females in preclinical models to capture sex-specific neuroimmune and glutamatergic adaptations.
- Electrophysiological studies should determine whether the microglial and structural alterations induced by chronic stress and cocaine are accompanied by changes in neuronal excitability and synaptic transmission within the NAc, and define the temporal window and mechanisms through which microglial modulation can restore glutamatergic balance and circuit function.
- Clinical trials assessing glial-targeting interventions, stratified by stress history and sex hormone status.
- Early intervention strategies for stress-exposed populations aimed at modulating neuroimmune and glutamatergic mechanisms before maladaptive plasticity becomes established.

## Figures and Tables

**Table 1:** Astrocyte–Microglia Crosstalk in Neuroimmune Regulation of Glutamatergic Signaling: Core Molecular and Behavioral Alterations.

Neuroglial Signaling	Stress- and/or Drug-Induced Molecular and Behavioral Outcomes	Key References
**Glutamate**	Stress-induced basal glutamate elevation and GLT-1 downregulation → glutamate spillover, a prolonged decay time of NMDA receptor-mediated currents → facilitation of cocaine intake.	[84,130]
	Cocaine-induced decreases in basal glutamate, reduced Xc^−^ activity and GLT-1 downregulation → potentiation of cocaine-evoked glutamate release → promotion of cocaine-seeking behavior	[159,160,161]
**Microglia**	Stress-induced microglial hyper-ramification/↑ Iba-1 expression.	[227,280,286]
	Drug-induced inflammatory pathways → microglial hyper-ramification/activation.	[269,271,290]
	Stress-induced microglial hyper-ramification/activation → facilitation of cocaine intake.	[85]
**Astrocyte**	Increased reactivity by non-contingent drug exposure	[308]
	Decreased GFAP expression by drug self-administration	[312,313]
	Decreased GFAP expression by stress/facilitation of cocaine intake	[85,315]
**Interaction Microglia–Astrocytes**	Stress- and Drug-Induced TNF-α/IL-1β elevation and behavioral consequences	[27,231,232,271,280]
	NF-κB pathway induction by drugs and control cocaine seeking	[360,361]
	Stress-induced NF-κB Pathway activation and facilitation of cocaine intake	[176]
	Stress-induced microglia–astrocyte remodeling, glutamate alterations, TNF-α elevation and escalation of cocaine intake	[85]
**Glutamate-** **Neuroimmune**	Glial regulation of glutamate homeostasis → control of stress- and drug-related responses.	[17,23,24]
	Glutamate-driven microglial activation induced by stress → facilitation of cocaine intake.	[85]

**Table 2:** Glial Regulation of Stress- and Drug-Induced Structural Synaptic Plasticity in the NAc: Synaptic Remodeling and Behavioral Changes.

Synaptic Remodeling	Stress- and/or Drug-Induced Synaptic Changes and Behavioral Outcomes	Key References
**Spine density changes**	Cocaine increased dendritic spine density	[382,383,384,385,386]
	Stress induced changes in total and mushroom spines and facilitation of cocaine intake	[84,151,152,365,393,394]
	Stress-induced microglial remodeling, dendritic spine alterations, and stress-related behaviors	[376]
**AMPAR surface expression**	Cocaine increased AMPA receptor trafficking and cocaine-induced behaviors	[387,390,391,392]
	Stress induced AMPAR upregulation, cross sensitization and facilitation of cocaine intake	[129,152]
	Microglia-driven AMPAR accumulation-mediated cocaine psychomotor activation	[378]
**Microglial pruning**	Microglia regulated synaptic remodeling	[189]
**TNF-α and/or IL-1β induced synaptic changes**	TNF-α controlled synaptic strength	[403]
	TNF-α regulated AMPAR trafficking and homeostatic synaptic scaling	[209,210]
	IL-1β altered bidirectional synaptic plasticity, AMPAR phosphorylation, and synaptic stability	[405,407,409]

**Table 3:** Repurposed Glutamatergic Therapies for Substance Use Disorder (SUDs) Comorbidity: Key Preclinical and Clinical Findings.

Agent	Mechanism of Action	Efficacy in Cocaine Use Disorder (SUDs)/Stress Comorbidity	Key References
**NAC**	Cystine–glutamate antiporter stimulation	Reduces cocaine-induced sensitization and relapse-related behaviors	[159,413,414,418]
	Can enhance GLT-1 expression/function	Decrease stress-induced cocaine seeking and reinstatement	[416]
	Neuroimmune regulation	Attenuates relapse vulnerability (clinical evidence)	[420,421,422,423,424]
		Reductions in PTSD symptoms and craving (clinical evidence)	[426]
**Ceftriaxone**	Enhance GLT-1 expression/function	Attenuates cocaine sensitization and drug seeking	[161,433,436]
		Prevents cross-sensitization and facilitation of cocaine self-administration	[84,151]
		Not tested clinically for comorbid stress-SUDs	
**Minocycline**	Inhibition of microglial activation	Attenuates microglial remodeling, neuroimmune, glutamate alterations and facilitation of cocaine intake	[85]
		Tested clinically for stress-related disorders (not SUDs)	[449,450]
**AMP/SUL**	Synergistic effects enhancing GLT-1 expression	Reduces cocaine reinstatement (not evaluated under stress)	[459,460]
		Not tested clinically for comorbid stress-SUDs	

## Data Availability

No new data were created or analyzed in this study. Data sharing is not applicable to this article.

## Abbreviations

The following abbreviations are used in this manuscript:

AMP/SULB	Ampicillin/Sulbactam
GFAP	Glial fibrillary acidic protein
GLT-1	Glutamate transporter-1
IL-1β	Interleukin 1 beta
LTP	long-term potentiation
LTD	long term depression
mGluR2/3	presynaptic metabotropic glutamate receptors 2/3
MSNs	Medium Spiny Neurons
NAC	N-Acetylcysteine
NAc	Nucleus Accumbens
N-myc	N-myc proto-oncogene protein
PTSD	Post-Traumatic Stress Disorder
SUDs	Substance Use Disorders
TNF-α	Tumor Necrosis Factor-alpha
